# The feasibility of using the *Körperkoordinationstest fur Kinder* (KTK) in a U.S. elementary physical education setting to assess gross motor skills specific to postural balance

**DOI:** 10.3389/fspor.2023.1133379

**Published:** 2023-05-22

**Authors:** Daryl Campbell-Pierre, Deborah J. Rhea

**Affiliations:** Department of Kinesiology, Texas Christian University, Fort Worth, TX, United States

**Keywords:** physical education, postural balance, children, KTK, sedentary, assessment, coordination

## Abstract

**Introduction:**

For the past ten years, falls have been the leading cause of nonfatal injuries for all age groups less than 15 years old. A significant rise in childhood sedentary behavior in schools and limited opportunities to be outside has led to motor coordination deficits which have contributed to fall injuries.

**Method:**

A German assessment tool, the *Körperkoordinationstest fur Kinder* (KTK), which has been used for decades in Western European countries, allows researchers and physical education teachers to evaluate typical and atypical children's motor coordination competencies related to dynamic postural balance successfully. No research has been published on the use of this assessment tool in the United States. If its use were found to be feasible in this country for identifying motor coordination deficits in typical and atypical children, it would close the gap in determining motor coordination. Therefore, this study sought in Phase 1 to determine the feasibility of using the *KTK* assessment in U.S. children and Phase 2 sought to determine the adaptability of the scoring protocol from use in other countries to the United States.

**Results:**

The Phase 1 results revealed the KTK assessment was feasible to administer in U.S. physical education class by addressing three challenges for U.S. schools: 1) KTK implementation, 2) time to assess each skill, and 3) the equipment availability and cost to implement the test in a physical education setting. In Phase 2, the researchers were able to determine the raw scores and motor quotient scores in this population and then were able to show similar scoring trends between U.S. children and Flemish children from a previous study.

**Conclusion:**

This assessment tool was deemed feasible and adaptable which is the first step to use the KTK in U.S. physical education elementary school settings.

## 1. Introduction

According to the Centers for Disease Control and Prevention (CDC), an estimated 9.2 million children annually have had an initial emergency room visit for an unintentional injury (1). Approximately 2.8 million of those children had the initial emergency visit due to fall injuries. For the past ten years, falls have been the leading cause of nonfatal injuries for all age groups less than 15 years old (1). A significant rise in childhood sedentary activity and limited opportunities to be outside have led to motor coordination deficits which has contributed to fall injuries (Xiang et al., 2020) (2). Children spend at least 7–8 h daily in a school setting which should lead to plenty of active time throughout the day. Sadly, adults have put a higher priority on the academic skills of children, placing physical education and recess as minimal offerings in schools (3). For children who receive recess during their seven-to-eight-hour school day, the majority see no more than 20 min daily (4).

Thirty years ago, recess was treated differently. It was recognized as an essential time for children to develop all dimensions of self (physical, mental, emotional, and social). Recess was always described as unstructured and outdoors where children had free choice of what they did at play, without teacher influence (5). It was the only way children engaged in play (6–8). Teachers supervised safety on the playground but did not interfere in the children's engagement in play. The value of play was recognized and given as a child's right (9), Article 31. Over the last few years, standardized tests have become the only marker of a child's success, leaving the best parts of a child behind in schools (10).

Much of the research has been focused on the detrimental effects of less play and recess on whole child development (11). Offering recess in schools allows children to reboot their brains for learning (12), decrease distress and anxiety (13) (Ordonez, 2020), and improve attentional focus (Rhea and Rivchun, 2018). Unstructured, outdoor recess allows children to meet the CDC's (1) recommended 60 min of physical activity daily which promotes healthy bodies and brain development (14, 15). Recess also boosts gross motor skill development through the engagement of running, jumping, swinging, and climbing, to name a few. These skills are highly beneficial for proper falling mechanics, motor skill proficiencies, and injury prevention (6, 7, 16).

Due to the decline of recess and unstructured, outdoor play opportunities in schools, physical activity researchers and physical educators have contributed this decline to motor skill deficiencies observed in physical education settings today (17). Researchers are realizing a strong parallel between children who have had outdoor play opportunities and their ability to be more proficient on motor skills in the physical education setting (16). Physical education (PE) teachers have recognized the decline in a child's ability to navigate different surfaces, coordinate motor skill movements, and fall in ways that prevent injuries (18). As the child ages, the alarming gap in skill proficiencies and number of injuries grows, especially as sport becomes the primary focus by middle school (19). At the root of these issues is whether a child can demonstrate the ability to coordinate muscle sequencing that preserves stability and postural balance also known as motor coordination which is key to motor skill proficiencies and everyday movements (20) (Pellegrini and Bohn-Gettler, 2013).

Balance, categorized as static or dynamic, is the act of maintaining, achieving, or restoring a base of support during any posture or activity (Ludwig et al., 2020). Both types are essential for a child to have the ability to maintain body control during motor skill tasks (21). Static balance is the ability to create a base of support during stationary tasks, whereas dynamic balance is the ability to create a base of support during tasks while moving (Conner et al., 2019). Developing dynamic balance proficiencies through recess and physical education are very important to master since they are predictors of a successful postural balance transition from childhood to adulthood (O'Brien et al., 2019). Dynamic balance unlocks a child's ability to perform functional activities of daily living that require maintaining a stable position, like grooming, dressing, walking down a hall in school, navigating uneven surfaces on the playground, and sitting at a desk to engage in classroom activities (22). *Assessing dynamic balance through gross motor coordination skills is needed to ensure c*hildren can engage in their environment across tasks effectively while limiting their risk of falling and preventing injuries inside and outside the school setting (23).

The physical education environment is the gold standard setting to evaluate school age children's gross motor coordination skills using appropriate assessment tools available to them (Loprinzi et al., 2015). Three issues need to be addressed when choosing an assessment tool for a physical education setting: (1) identifying the most appropriate assessment tool to use for the type of skill proficiency needed; (2) the person assessing the skills should be able to administer it accurately; and (3) knowing how to incorporate it with any physical education class size quickly and efficiently (24). A motor coordination assessment must incorporate multiple fitness concepts that align with dynamic postural balance movements like strength, speed, endurance, and flexibility (21). Most motor coordination assessments are used in a clinical setting to diagnose individual motor deficiencies. If physical educators are going to use a motor coordination assessment, it must evaluate dynamic components of postural balance with the goal of preventing fall related injuries and be feasible in a variety of physical education settings (Cadore et al., 2013).

Several motor coordination assessments are used in the United States to assess a single fitness component related to balance and coordinative capacities rather than multiple fitness components related to dynamic balance (1). Researchers have found it difficult to label one assessment as the best motor coordination tool (Bardid et al., 2016). The Movement Assessment Battery for Children (M-ABC) is a motor coordination assessment designed to detect and evaluate children's functional movement skill (FMS) development deficiencies (Johnston, 2016). The M-ABC evaluates fine and gross motor skills within the scope of the assessment's evaluation tasks (Wuang et al., 2009). Fine motor skills are essential to determining a child's manual dexterity but do not specifically address the motor skills needed to establish postural balance capabilities. Not only does it lack needed criteria, but it also takes longer per child to administer, i.e., 20–40 min, than is suitable for different physical education class sizes. Therefore, the M-ABC would not be the most appropriate assessment for physical educators to evaluate motor skills needed to assess only dynamic balance in children. The Brunininks-Oseretsky Test of Motor Proficiency (BOTMP) is another common motor coordination assessment designed to evaluate motor skill deficiencies in school-age children due to comorbidity from a specific diagnosis like cerebral palsy, developmental coordination disorder, and autism (26). This motor coordination tool would not be the most inclusive assessment for physical education classes because it was designed to target atypical children defined as children with motor deficiencies. Physical education classes in the U.S. include very diverse developmental levels of children ranging from typically developing to atypically developing. In addition, the BOTMP generally takes about 60 min to administer to one child (27). That is not appropriate with the profile of physical education classes usually ranging from 50 to 60 children and have limited time of 35–45 min per class period on average. Therefore, the BOTMP would also not be an appropriate assessment for this study. Unfortunately, limited motor coordination assessments exist in the U.S. designed to evaluate gross motor skills that are directly related to dynamic postural balance, focus on inclusivity, and meet the physical education class time constraints. This led to examining an assessment tool commonly used outside of the U.S. that meets these challenges.

The *Körperkoordinationstest fur Kinder* (KTK) (Bardid et al., 2015) is a German developed assessment tool focused exclusively on gross motor skills that take approximately 15–20 min per child. The KTK was designed to evaluate multiple fitness components, i.e., agility, speed, balance, strength, and coordination, that align specifically with postural balance skills (Nascimento et al., 2018). No U.S. studies have been documented that use the KTK, but studies from other countries like Germany, Finland, Brazil, and England have shown the KTK to be highly effective in elementary school aged children (28). As a result, researchers from many other countries have adopted the KTK to assess children's gross motor skills and postural balance abilities in PE classes (28). The KTK allowed these researchers to evaluate typical and atypical children straightforwardly and objectively with limited interference of other physical fitness components outside of the key four elements of strength, speed, endurance, and flexibility. In addition, the KTK has a 90% validity rate in identifying children with underlying cognitive impairments in conjunction with gross motor skills (29). This illustrates the KTK assessment as one of a kind in its ability to connect gross motor skills directly to postural balance and cognitive abilities in children (1).

Moreira et al. (2019) deemed the KTK assessment viable for research, professional practice, and educational settings. Many researchers have found the KTK to be a valid and adequate tool to assess motor coordination in children between the ages of 5–14 in comparison to the M-ABC and BOTMP motor coordination assessments that are widely used in western European countries (30) (Zoia et al., 2018). The KTK assessment has also been used with a variety of children's populations who have motor performance deficits. The flexibility of this assessment is designed to allow researchers and physical educators to evaluate multiple children within a physical education class who present as typically or atypically developing to establish a motor competence baseline. All indicators show U.S. physical educators should be able to adopt the KTK to assess and evaluate the multiple fitness components (i.e., agility, speed, balance, strength, and coordination) related to motor competence skills and postural balance in children through 14 years of age. The KTK would give physical educators an alternative way to identify development needs for children to reach age-appropriate motor abilities inside and outside the classroom and prevent injuries due to falls.

Although the KTK can be an ideal assessment tool to evaluate postural balance in schools, the assessment still presents some challenges that PE teachers must overcome for the assessment to be deemed feasible to use in a U.S. physical education setting. The three glaring challenges in the U.S. that may not be relevant in other countries are (1) implementation of the KTK assessment (2) time to assess each skill; and (3) equipment availability and cost to run the tests.

Therefore, Phase 1 of this study was to determine the feasibility of using the *Körperkoordinationstest fur Kinder* (KTK) assessment to evaluate gross motor coordination skills specific to postural balance in American children. The feasibility was determined by addressing each of the challenges spelled out above. Phase II was to determine the adaptability of the scoring protocol from use in other countries to the United States. First, raw scores and motor quotient scores would be established through the subtest assessments and then will be compared between 8 and 10-year-old U.S. children who were assessed in physical education classes from this study with 8–10-year-old Flemish children from the 2008 suitability study (1).

## 2. Phase 1 assessment preparation, subtest descriptions, and feasibility challenges

The KTK assessment preparation processes were developed to assure all researchers and school personnel involved in the KTK assessment knew the implementation processes, how to set up the subtests, and the assessment procedures before administering the KTK assessment. The KTK feasibility was addressed through identifying three implementation challenges associated with the KTK assessment novel to a U.S. population. The first challenge addressed implementation processes: (1) number of teachers needed to monitor each subtest, (2) identifying the set up and rotation subtest procedures, and (3) determining the number of children who can engage in each subtest at one time. The second challenge addressed the time it would take to assess each skill. The third challenge addressed the equipment availability and cost to implement the tests in a gym setting.

### 2.1. Pre-KTK assessment preparation processes

The KTK is comprised of four subtests: Walking Backwards (WB), Lateral Jumping (LJ), Sideways Stepping (SS) and Single Leg Hop (SH). To assess whether KTK implementation is feasible in a U.S. physical education setting, the PI and research team needed to provide additional information to the physical educators to ensure a fluid and consistent evaluation of the assessment. Therefore, an initial meeting was scheduled with the physical education teachers at the school gymnasium where the subtests would be set-up. The meeting, about an hour in length, consisted of explaining the benefits of the KTK, providing the procedures for each subtest, and demonstrating how to implement each subtest correctly in the gym setting. Physical educators were able to practice each subtest and ask any questions related to KTK equipment set up and implementation.

A second meeting, also about an hour, was scheduled two weeks later to demonstrate each KTK subtest again as well as introduce the subtest score sheets to assure that each PE teacher was competent in assessing and scoring their children correctly. Instructions and tips about how to rotate, monitor, and score each subtest were explained. The physical education teachers gave the PI and two other researchers a tour of the gym to develop an equipment setup strategy to ensure child's safety at each of the subtest stations. After the teachers felt comfortable with the implementation and scoring processes at this meeting, the data collection days were scheduled for the following week.

The next step that had to be set up prior to the day of arrival was children's information organization and post testing feedback. The researcher requested and organized the children's rosters for each grade level into four equal groups depending on the number of children assigned to each class. The teachers were each given a master score sheet for each grade level on the first implementation day that had the children assigned to their station listed first followed by the children who would rotate to them sequentially throughout the two-day process listed next in order. Once each child completed a subtest the teacher would direct the child to the next subtest in a continuous manner until time expired for that class. If a child's name did not appear on the score sheet, then the teacher could simply write in their name to complete the evaluation process.

After each class period was complete, the PI met with the evaluation team to discuss the evaluation process and areas of improvement. The PI would document the percentage of completed subtests, the time taken to complete the subtest, the resources needed to complete each subtest, and the equipment to set up the subtest.

### 2.2. KTK subtest descriptions, equipment needs, and scoring

In order to assure similar KTK assessment implementation, the KTK manual was acquired to substantiate the KTK subtest descriptions, equipment specifications, and scoring information.

#### 2.2.1. Subtest 1: balance beam (WB)

##### 2.2.1.1. Description and equipment information

This subtest measured balance, rhythm, and strength. All three of these physical skill components determine the child's ability to maintain postural stability while walking backwards on three different widths of balance beams. Balance beam one is 6.0 cm in width, followed by balance beam two which is 4.5 cm in width, and finally balance beam three which is 3.0 cm in width. All three balance beams are seven feet in length.

###### 2.2.1.1.1. Scoring Instructions

Children remove their socks and shoes before participating in the WB subtest. Children were instructed to walk backward on the three width-size balance beams (6.0 cm, 4.5 cm & 3.0 cm). The children were then asked to take a maximum of eight steps per trial, for a maximum of 24 steps per balance beam, with a total of 72 steps for the three different balance beams. Each child has three attempts per balance beam to reach the maximum of eight steps per beam. The child starts the evaluation process by walking up the balance beam until they reach the wooden platform. Once the child reaches the wooden platform, the backward walking assessment begins. The first step from the wooden platform onto the balance beam backwards is called the plantar step. This step does not count towards the eight steps possible per trial. One successful step backward equals one point for the possible eight points per trial. If a child falls off of the balance beam or any body part touches the ground while on the balance beam, the child must restart on the wooden platform for their next trial attempt. Once a child completes their three attempts, they transition to the next balance beam size to demonstrate the same procedures**.**

#### 2.2.2. Subtest 2: lateral jumping (LJ)

##### 2.2.2.1. Description and equipment information

This subtest measured speed, rhythm, and agility. All three of these physical components will determine the child's ability to move laterally and maintain postural stability while jumping back and forth repeatedly for 15 s. The lateral jump is assessed using a wooden obstacle measuring 5 ft 10 inches long by 24 inches wide with a flat wooden surface vertically positioned that is 25 cm long, 25 cm wide, and 5.7 cm high.

###### 2.2.2.1.1. Scoring Instructions

Children were instructed to stand on the right side of the wooden divider with both feet flat on the ground before the lateral jump activity occurs. The child is tasked with jumping laterally over the wooden divider as many times as possible for 15 s. Children must successfully not touch the wooden divider and have both feet touch the ground simultaneously during their lateral jump attempt for the point to count towards their final score. Each successful jump counts for one point toward their final score. Due to this subtest being a timed test, there is no total maximum number. The child must try their best to reach their maximum number of successful jumps in the 15 s window. Each child had two attempts to complete the LJ subtest to populate a total sum score from the two attempts.

#### 2.2.3. Subtest 3: sideways stepping (SS)

##### 2.2.3.1. Description and equipment information

This subtest assesses how well children move sideways on a wooden platform using a repetitive crossover motion. The SS subtest measured speed, rhythm, strength, and balance. All three of these physical components will determine the child's ability to move laterally and maintain postural stability while stepping sideways repeatedly for 20 s. The sideways step subtest requires two wooden platforms with dimensions of 25 cm × 25 cm × 5.7 cm (*L* × *W* × *H*).

###### 2.2.3.1.1. Scoring Instructions

Children were instructed to stand on one wooden platform before starting the SS subtests. The child would pick up the second wooden platform to the left or right of them and perform a crossover maneuver to the other side of their body. The child would then step sideways onto the platform and perform the crossover maneuver repeatedly for 20 s to determine how many times the child could step onto a new platform and do the crossover maneuver. The children were given two attempts on this subtest. A 10 s break must be given between each attempt to allow the child to regain composure before the next attempt. One point was awarded to the child for performing the crossover maneuver (1 pt. = Crossover), and one point was awarded for stepping onto the wooden plank after the cross maneuver was performed (1 = stepping onto the new platform). This is a timed subtest, so there is no preset maximum number of steps, so each child is encouraged to do their best. After both attempts were completed, then the sum of both attempts was documented.

#### 2.2.4. Subtest 4: single leg hop (SH)

##### 2.2.4.1. Description and equipment information

This subtest assesses each leg individually to hop over a foam obstacle with an increasing height of 5 cm per successful hop. This subtest measured strength, rhythm, and balance. All three of these components work together in a synergy pattern to identify the explosiveness of a single leg to clear a successful landing over increased height of the foam pad. As children clear the height of each foam pad, the evaluator will add 5 cm foam pad increments until they can no longer clear the height over three attempts. The Trifold mat is a requirement for children to complete this jumping subtest. The evaluator is responsible for ensuring the child has a clear pathway of at least six feet of space to ensure child safety. Twelve foam pads with a depth of 5 cm each are needed to implement this subtest. The dimension of the foam pad is 60 cm × 20 cm × 5 cm (*L* × *W* × *H*). The goal is to clear all 12 foam pads stacked on top of each other (60 cm in height total) with each leg.

###### 2.2.4.1.1. Scoring Instructions

Children removed their socks and shoes before participating in the jumping for height activity. The child stands off the trifold gym mat before starting the assessment. The child must be instructed on which leg is being evaluated then the child balances on that specific leg (right or left) before trying their first attempt over the foam obstacle. The child must hop over the foam obstacle from the evaluated leg, land on the same leg, and complete two hops after landing to receive a point-worthy score. Suppose the child had any other body part hit the ground, especially the non-evaluated leg or could not complete two additional hops. In that case, the point score does not count. Children will be given three attempts per leg (right & left) to hop over the foam obstacle. Points for this subtest are determined by what attempt the child can successfully hop over the foam obstacle. Three points are awarded to the child if they can complete the hop on the first attempt (1st = 3 points), two points are awarded if the child completes a successful jump on the second attempt (2nd = 2 points), and one point is awarded if the child can complete a successful jump on the third and final attempt (3rd = 1 point). After one leg is complete then the evaluator will complete the same scoring procedure for the opposite leg. After each leg has been evaluated, the evaluator will increase the height of the foam obstacle in increments of 5 cm up to 60 cm total (5 cm, 10 cm, 15 cm, 20 cm, 25 cm, etc.) For example, suppose the child is not able to successfully jump over the foam obstacle in three attempts. In that case, they will receive a score of zero and indicate to the evaluator that they have reached their JH ceiling. The child will not be able to move forward in height with that specific leg. Once both legs have been eliminated by not reaching the three-attempt threshold, the evaluation for the child must be stopped and the total score must be summed together. The child can reach a maximum of 72 points for this subtest if the child is able to successfully hop over each foam obstacle on their first attempt to receive a score of three for each of the twelve obstacles.

### 2.3. Feasibility challenges

Next, was to address challenges we felt would prevent the KTK from being feasible in physical education classes from the U.S.

#### 2.3.1. KTK implementation

The first challenge, implementation, included three parts: (1) the number of evaluators needed to monitor each subtest; (2) identifying the set up and rotation subtest procedures, and (3) determining the number of children who can engage in each subtest at one time.

##### 2.3.1.1. Evaluator needs

The PI scheduled one researcher or teacher to administer and assess each of the four subtests. The evaluation team consisted of the PI and one additional member of the research team along with two physical education teachers.

##### 2.3.1.2. Subtest procedures

The PI and teachers arrived 30 min before the first physical education class was to arrive to set up the equipment, provide the score sheets with the children's rosters included, clipboards and pencils, and review subtest responsibilities for each evaluator. One gym (approximately 120 feet long by 75 feet wide) was used to complete the subtests. Most elementary school gyms in the U.S. are not this big, but even for the smallest of gyms, there is still plenty of space to have all four subtests set up simultaneously. At this school, though, it was easy to set up *the stations far enough apart to address any safety concerns and not overwhelm an area with too many children. Each subtest needed to have about 8 feet of space to execute all protocols for each subtest completely.* The PI administered the equipment set-up and execution of the subtests along with the physical educators. After completing the KTK evaluation the PI had a brief meeting with each evaluator to ask if there were any questions regarding the evaluation process before children arrived for class.

##### 2.3.1.3. Number of manageable students

*Since only one set of equipment was used at each station, the children were divided equally into four groups and assigned to one of the four subtests. Each of the grade levels had 20–35 children, therefore a station could have anywhere from 5 to 9 children depending on the grade level class size.* For example, if a class had 20 total children, the class would be evenly distributed with five children going to each of the four stations. *The plan was for each child to rotate chronologically to the next subtest as they completed a subtest. The plan was that two subtests would be completed by all children on the first day and the other two subtests would be completed on the 2nd day.* Each physical education grade level had a varied time schedule. The 3rd graders had 40 min, the 4th graders had 35 min, and the 5th graders had 45 min. Although each grade level had varied minutes and number of children, the PI felt the two day schedule could still work because the smallest number of children (20) was linked to the grade with the lowest number of class time minutes (35) The largest class sizes had the most minutes of class time, so we felt the class sizes with the number of minutes would work out the same for all three grade levels. A third day would be used to perform any make-up subtests for children who were absent during one or both days of evaluation.

After completing all subtests for the three grade levels, the evaluators reflected on how well the KTK implementation worked overall. First, the equipment set up took approximately 30 min prior to a class arriving. So, equipment set up should take place at least 45–60 min prior to the first-class arrival so there is sufficient preparation time prior to child entry. Second, the setup of two subtests on the right side and two subtests on the left side of the gym was feasible with four evaluators. The physical educators felt the subtest implementation and scoring could be accomplished with two to three evaluators per class, depending on the class size, the class minutes, and the amount of equipment available for the teacher to use in each class. This study showed that for a class up to 35 children, divided into no more than nine children per subtest group, it was feasible to execute the evaluation process, manage children, and be able to successfully move children to their next subtest without any downtime.

##### 2.3.1.4. Time to assess each subtest

The second challenge examined the amount of time it took to assess each subtest. This was evaluated by taking the number of minutes each physical education class period had with how many children were in each class, and then determining how long it took to evaluate the four subtests for each grade level. For example, the largest number of children (*N* = 37) was the 5th grade class which had 45 min classes daily. After monitoring the assessment to completion, it took 180 min or four 45 min physical education class periods to complete the subtests for 5th grade, but it took the same number of days for each of the other grade levels as well due to the lower number of class minutes daily. The time per child averaged to about four and half minutes to complete all four subtests. This time element was based on only using one set of equipment over the four days and one evaluator per subtest per day. After discussing this time element with the teachers, the consensus was introducing more sets of equipment for each subtest would aid in quicker subtest completion. We also realized that the balance beam and single leg hop subtests took longer to evaluate each child because the requirements for those two tests were more cumbersome than the other two tests. For example, the balance beam (WB) subtest had three trials for each level of completion and for the single leg hop (SH), it required three trials for each leg per level of completion. The other two subtests were for speed and agility, so they were set with a stopwatch and only needed two trials per subtest. If additional equipment is provided expressly for the WB and SH subtest, more children can be evaluated simultaneously for less overall completion time.

##### 2.3.1.5. Equipment availability and cost

The third challenge examined how to acquire the equipment and how much it would cost to purchase the equipment. No commercial manufacturer builds the three balance beams of differing sizes, sidestep boards, the lateral step single board, or the single leg hop pads that are required to assess the KTK subtests. The research team took the dimensions for each piece of equipment given in the equipment section above and hired a carpenter to make the equipment based on the specifications. Once built, each piece had to be sanded and painted to create smooth wood surfaces for safety and for the balance beams, they were painted different colors to represent the width differences. Pictures and videos were available to make sure the equipment we produced met the KTK equipment requirements and matched the other countries.

The wood for the WB, SS, and LJ subtest was purchased from a local hardware store for $150. The foam pads for the single leg hop subtest were created out of a pillow top cushion that cost $50. The labor charged for the whole set would probably vary by carpenter to build the different equipment pieces. Therefore, the equipment materials for this study cost $200 and the labor for our carpenter was $100 for a total of $300 to have one set of equipment made.

### 2.4. Phase 1 discussion

In order to determine feasibility of the *Körperkoordinationstest fur Kinder* (KTK), *preparing for the assessment and* substantiating the KTK subtest descriptions, equipment specifications, and scoring information had to be accomplished. *These steps were effectively completed. Teachers were engaged in the process of where to setup the subtests in the gym, how many children would be assessed, and how to score each of the subtests. This created a smooth transition to the feasibility phase which showed the KTK* was feasible to administer in an American physical education class. The three identified challenges the U.S. schools would face are (1) KTK implementation, (2) time to assess each subtest, and (3) the equipment availability and cost to implement the test in a physical education setting. The interpretation of how to set up the equipment, implement the subtests, score each subtest, and manufacture the equipment needed for each subtest went smoothly in this setting. This is very promising for implementation of this battery of tests in 5–14 age children.

Although one full set of the KTK subtest equipment was feasible to evaluate each grade level with smaller class sizes, i.e., 20–35, this study has shown at least two to three sets of some of the equipment may be necessary to accommodate larger numbers of children and complete the tasks in two to three days. Other researchers have communicated that it takes approximately 20–30 min per child to complete all four subtests (31). We found that it takes approximately 5 min per child to complete all four subtests if you have one piece of equipment. We feel the assessments can transition much quicker if more equipment is set up. We determined that that single leg hop subtest takes the most time because a child must do the right leg hop from 5 cm up to 60 cm, followed by the left leg hop from 5 cm to 60 cm prior to completing the subtest.

Anecdotally, we found that the children enjoyed participating in the four subtests. Multiple child comments were heard and non-verbal cues were seen while the children participated in the KTK subtests. They were excited, had smiles on their faces, challenged themselves to improve, and encouraged each other through the process. This could be a result of the KTK assessment being novel and fun. We observed that children did not feel that other children were watching them make a mistake when participating in the four subtests. Children did not know why the assessment was conducted and the instructions provided did not specify that the KTK was an assessment. The children were informed we were assessing balance, agility, and coordination. So, children treated the assessment as any other physical activity incorporated into their physical education class and did not seem to feel the pressure of failing when participating in the KTK assessment.

Lastly, teachers stated that they felt they could implement the subtests with success. They also loved that there was a meaningful outcome given for each subtest. The feedback derived from these scores would be very helpful for teachers to adjust the physical education curriculum to assist in postural balance development. Future studies will help determine if these challenges can be addressed more succinctly, but overall, the KTK is feasible to use in a United States physical education class.

## 3. Phase II determining adaptability of the KTK assessment

### 3.1. Participants

The participants for this study were children attending a North Texas school identified for the feasibility study. The children were between the ages of 8–10 and identified as either a boy or a girl to match the standardization profile of the norm scores. The children had the opportunity to engage in three 15 min outdoor, unstructured play breaks resulting in 45 min of recess along with a daily 35–45 min physical education class depending on the grade level. The children were identified as typically developing children and presented at different development levels during their physical education classes. All the children were able to follow instructions and had no glaring injuries that would prevent them from participating in the evaluation of the KTK assessment.

Table 1 provides the number and percentage of children involved in the KTK analyses by age and sex. A convenience sample of 82 boys and girls, ages 8–10 (grades 3–5), completed the KTK assessment. Inclusion criteria were parent consent, assent of each child, daily involvement in physical education and recess, and full use of their whole body to complete each physical component of the KTK subtest. The exclusion criteria were injuries preventing a child from executing a required body movement or a child's verbal refusal to participate at any time during the data collection process. Each child who met the inclusion criteria above was evaluated on all four KTK subtests. An additional 12 children did not complete all four subtests because of injury or refusal to participate and therefore were removed from the sample for the final total of 82.

**Table 1:** Participants by age and sex.

Age	Males 40 (%)	Females 42 (%)
8-years	12 (15.0)	13 (16.0)
9-years	10 (12.5)	10 (12.5)
10-years	18 (21.5)	19 (22.5)
Grand total	(48.7)	(51.0)

### 3.2. Procedures

The University Institutional Review Board approved the cross-sectional feasibility study design. One school in the North Texas region was identified to participate in this study who had at least 45 min of recess daily. The school administration team and physical education teachers at this school were asked to participate and approved prior to submitting letters to parents. Children were included who had parent consent but could decline participation at any time if they felt uncomfortable being evaluated. All these procedures were completed prior to the data collection phase.

Subtest Procedures. All subtest instructions were explained to all children at the beginning of the class period. After the instructions and demonstration were given, children were divided into even groups depending on their class sizes and each assigned subtest evaluator took their group of children to the subtest station. Each evaluator collected all scores on their subtest score sheet for each child. Once a subtest was completed for a whole group, the evaluator would direct the children to the next subtest for evaluation. At the end of each P.E. class, the PI collected all score sheets and secured them until the next evaluation day. Once each child finished all subtests, the PI collected all score sheets and completed a focus group session with the teachers.

### 3.3. KTK motor coordination ability scoring

The KTK was used to measure gross motor coordination abilities related to dynamic postural balance. The KTK manual provides the process to determine each child's motor coordination ability. Raw scores must be determined, followed by converting them to individual child motor quotients (MQ). The raw scores were created by adding the number of attempts together from each KTK subtest. The total MQ value was computed by adding the individual converted MQ values from the four subtests and then standardizing them by age and sex using Table 2 numbers provided below (32, 33). All four-subtests had to be completed for the raw scores to be converted to Motor Quotient scores. These MQ scores are required to determine motor coordination ability levels.

**Table 2:** Motor quotient conversion values.

Sum MQ1–MQ4	M–Q	Sum MQ1–MQ4	M–Q	Sum MQ1–MQ4	M–Q
215–217	40	341–343	81	468–470	122
218–220	41	344–346	82	471–473	123
221–223	42	347–349	83	474–476	124
224–226	43	350–352	84	477–479	125
227–229	44	353–355	85	480–482	126
230–232	45	356–358	86	483–485	127
233–235	46	359–361	87	486–488	128
236–238	47	362–364	88	489–491	129
239–241	48	365–367	89	492–495	130
242–244	49	368–371	90	496–498	131
245–248	50	372–374	91	499–501	132
249–251	51	375–377	92	502–504	133
252–253	52	378–380	93	505–507	134
254–256	53	381–383	94	508–510	135
257–259	54	384–386	95	511–513	136
260–262	55	387–389	96	514–516	137
263–265	56	390–392	97	517–519	138
266–268	57	393–395	98	520–522	139
269–271	58	396–398	99	523–526	140
272–274	59	399–402	100	527–529	141
275–278	60	403–405	101	530–532	142
279–281	61	406–408	102	534–536	143
282–284	62	409–410	103	537–539	144
285–287	63	411–413	104	541–543	145
288–290	64	414–417	105	544–546	146
291–293	65	418–420	106	547–549	147
294–296	66	421–423	107	500–552	148
279–299	67	424–426	108	553–555	149
300–302	68	427–429	109	556–559	150
303–305	69	430–433	110		
306–309	70	434–436	111		
310–312	71	437–439	112		
313–315	72	440–442	113		
316–318	73	443–445	114		
319–321	74	446–448	115		
322–324	75	449–451	116		
325–327	76	452–554	117		
328–330	77	455–457	118		
331–333	78	458–460	119		
334–336	79	461–464	120		
337–340	80	465–467	121		

Motor Quotients (MQ) scores. MQ1 = MQ for LJ; MQ2 = MQ = MQ for SS; MQ3 for WB; MQ4 = MQ for SH.

The total MQ identifies a child's motor coordination performance into five categories based on norms determined by age and sex. According to Kiphard & Schilling (33), children who have MQ values less than 70 are categorized as having severe gross motor coordination abilities. Children scoring between 71 and 85 have moderate gross motor coordination abilities, and children scoring between 86 and 115 have normal gross motor coordination abilities. Children scoring between 116 and 130 have good gross motor coordination abilities, and children scoring above or equivalent to 131 have great gross motor coordination abilities. Biino et al. (2022) reported that the KTK has an acceptable construct validity and a test-retest reliability score of 0.97. Each subtest item had a reliability coefficient ranging from 0.80 to 0.96.

### 3.4. Results

#### 3.4.1. Raw scores and motor quotient scores

The children in this study were scored on the four KTK subtests using the scoring protocol established in Phase 1 of this study. These scores were needed to determine the adaptability of the scoring protocol from use in other countries to the U.S. The U.S. research team revealed they were able to produce measurable raw scores from each KTK subtest. The raw scores were then converted to KTK motor quotient scores to determine each child's motor coordination ability (see in Table 3). Descriptive statistics were used to determine the means and standard deviations for the total sample by age and sex which can be seen in Table 3.

**Table 3:** American MQ values by age and sex.

	8-year-olds				9-year-olds				10-year-olds			
	*N*	Male	*N*	Female	*N*	Male	*N*	Female	*N*	Male	*N*	Female
WB	13	100.5 ± 13.42	12	99.9 ± 16.23	10	86.8 ± 14.29	10	89.6 ± 15.25	19	91.2 ± 19.35	18	94.83 ± 12.55
SH	13	104.1 ± 15.04	12	114.7 ± 17.08	10	99.8 ± 18.56	10	100.0 ± 12.77	19	105.2 ± 15.56	18	105.50 ±10.14
SS	13	88.5 ± 14.11	12	95.2 ± 12.94	10	87.3 ± 13.58	10	95.2 ± 18.82	19	97.2 ± 20.78	18	77.83 ± 9.79
LJ	13	115.4 ± 17.43	12	118.7 ± 17.71	10	117.7 ± 17.70	10	101.5 ± 24.79	19	125.0 ± 17.10	18	95.70 ± 12.90
Total by Sex	13	102.1 ± 15.00	12	107.1 ± 15.99	10	97.9 ± 16.03	10	96.6 ± 17.91	19	104.7 ± 18.19	18	93.46 ± 11.34
Total by age	25	104.62 ± 15.49			20	97.23 ± 16.97			35	99.05 ± 14.76		

#### 3.4.2. Motor coordination performance levels

**Figure 1:**
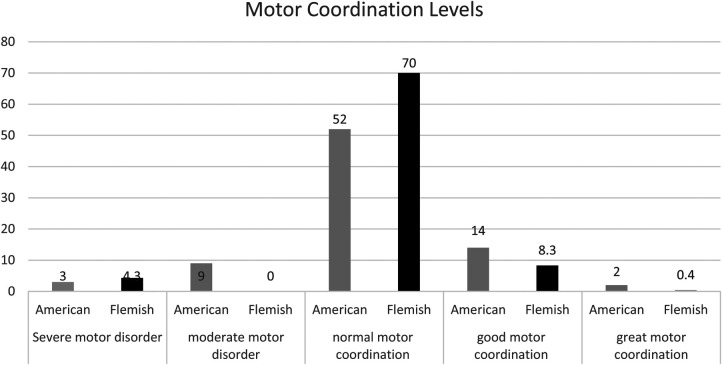
American and Flemish motor coordination levels determined by the KTK.

The Motor Quotients for the entire sample in Figure 1 revealed 15% of the American 8, 9 and 10-year-old children presented with motor problems (severe and moderate) according to the MQ values, 65% of the total sample presented with normal gross motor coordination ability, and 20% of total sample presented with good to great gross motor coordination according to the manual. The Flemish 8, 9 and 10-year-old children reported similar trends with 21% of their total sample identifying as having severe or moderate motor problems, 70.2% of their total sample presented as having normal motor coordination ability, and 8.7% of their total sample presented with good to great motor coordination.

#### 3.4.3. American/Flemish MQ similar trends

Means and standard deviations of American and Flemish children's total MQ values for each of the four KTK subtests are reported in Tables 4 below by age and sex. Similar trends can be seen across each of the subtests. The scoring was similar by sex and age for the majority of subtests. For example, the nine-year-old and ten-year-old American and Flemish groups had similar trends of females performing better than the males on the WB subtest and males performing better than females on the LJ subtest. The two subtests that had a little more fluctuation between the two groups were SH and SS and mainly with the eight-year-olds. There was not enough fluctuation for any of the subtest scores to warrant any scoring concerns with the U.S. population.

**Table 4:** Flemish MQ values by age and sex.

Flemish participants motor quotient values												
	8-year-olds				9-year-olds				10-year-olds			
	*N*	Male	*N*	Female	*N*	Male	*N*	Female	*N*	Male	*N*	Female
WB	238	87.95 ± 15.17	248	91.49 ± 13.71	279	88.7 ± 13.73	266	91.6 ± 14.65	147	89.67 ± 13.40	212	91.93 ± 13.14
SH	238	105.1 ± 12.78	248	105.82 ± 14.14	279	105.24 ± 11.57	266	97.99 ± 13.28	147	104.49 ± 11.91	212	94.37 ± 13.77
SS	238	93.01 ± 14.35	248	93.95 ± 13.28	279	92.3 ± 14.19	266	92.29 ± 13.21	147	88.48 ± 11.99	212	88.52 ± 13.34
LJ	238	105.6 ± 14.39	248	105.61 ± 12.81	279	108.62 ± 12.11	266	94.72 ± 15.79	147	103.48 ± 13.06	212	93.12 ± 13.95
Total by gender	238	97.19 ± 14.83	248	98.56 ± 13.87	279	98.22 ± 13.08	266	92.34.3 ± 14.91	147	95.36 ± 12.55	212	89.50 ± 13.60
Total combined	486	97.87 ± 14.37			545	95.21 ± 14.34			359	92.96 ± 13.29		

### 3.5. Phase II discussion

The purpose of phase II was to determine the adaptability of the scoring protocol from use in other countries to the U.S. This study's research team demonstrated they could score each KTK subtest. These individual scores were then able to produce measurable raw scores that could be converted to KTK motor quotient scores to determine their children's motor coordination abilities. This research team followed the scoring protocol established in Phase 1 to produce these results, indicating that the scoring protocol is adaptable to use in a U.S. physical education setting.

The KTK also connects the child's cognitive abilities to their motor abilities. The motor coordination abilities of the U.S. children revealed that even when the schools provided 45 min of recess daily and daily physical education classes, 15% of these children presented with severe and moderate motor coordination abilities. On average, the additional opportunities for physical activity throughout their school day are more than most children receive in most school districts in the U.S. Because recess has diminished from U.S. schools for the past ten years, some of the subtest scoring differences may be because of the lack of movement and increased sedentary behaviors. The KTK showed to be highly effective in identifying children's motor ability differences. These results allow researchers and physical educators to examine a child's motor strengths and weaknesses as the child focuses on tasks they find fun and engaging instead of arduous.

The final step for these results was to examine similar trends between the U.S. children and the Flemish children by following the protocol provided in the implementation section in Phase 1, utilizing the scoring instructions for each subtest, and using the replicated subtest equipment with our American children in a physical education setting. The trends were very similar for the most part. Further investigation would be interesting to determine why differences might occur between children from different countries. The differences could be caused by the amount of movement each group receives daily or how much class time is spent on performing these movements. However, similar trend analyses did reveal that the scoring protocol was adaptable to use in a U.S. physical education setting because we could generate raw scores by following the implementation criteria. Once the raw scores were generated, the MQ values were able to be determined and ability levels were identified based on the KTK standardized norms. Establishing an assessment tool of this caliber in the U.S. will not only identify dynamic postural balance weaknesses and strengths through motor competence deficiencies, but it could also aid physical education teachers in the process of reducing the child injury rates due to falling. This type of motor identification could benefit physical educators in their curriculum development process to target specific skills needed for each grade level depending on their class performance and aid in motor coordination support for atypically developing children.

Establishing an assessment tool of this caliber in the U.S. will not only identify dynamic postural balance weaknesses and strengths through motor competence deficiencies, but it could also aid physical education teachers in the process of reducing the child injury rates due to falling. This type of motor identification could benefit physical educators in their curriculum development process to target specific skills needed for each grade level depending on their class performance and aid in motor coordination support for atypically developing children.

## 4. Limitations

One limitation is the manual needed to be translated from Dutch to English so some of the elements might have been misinterpreted for the standardized procedures necessary to administer the assessment. We have not developed a standardized procedure for the KTK assessment. Therefore, the structure of how the assessment can be administered could change in future studies to fit the demands of a physical education classroom.

## Data Availability

The original contributions presented in the study are included in the article, further inquiries can be directed to the corresponding authors.

## References

[1] Centers for Disease Control and Prevention. Children safety and injury prevention: 2019 overview. Atlanta, GA: U.S. Department of Health and Human Services (2019).

[2] DelCastillo-Andrés Ó Toronjo-Hornillo L Rodríguez-López M Castañeda-Vázquez C Campos-Mesa MDC. Children’s improvement of a motor response during backward falls through the implementation of a safe fall program. Int J Environ Res Public Health. (2018) 15(12):2669. 10.3390/ijerph15122669 30486425 PMC6313405

[3] Brusseau TA Hannon JC. Impacting children's health and academic performance through comprehensive school physical activity programming. Int Electron J Elem Educ. (2015) 7(3):441–50.

[4] Ramstetter C Murray R. Time to play: recognizing the benefits of recess. Am Educ. (2017) 41(1):17.

[5] Bauml M Patton MM Rhea D. A qualitative study of teachers’ perceptions of increased recess time on teaching, learning, and behavior. J Res Child Educ. (2020) 34(4):506–20. 10.1080/02568543.2020.1718808

[6] Lee J Zhang T Chu TL Gu X. Effects of a need-supportive motor skill intervention on children’s motor skill competence and physical activity. Children. (2020) 7(3):21. 10.3390/children7030021 32192010 PMC7140861

[7] Lee RLT Lane S Brown G Leung C Kwok SWH Chan SWC. Systematic review of the impact of unstructured play interventions to improve young children's physical, social, and emotional wellbeing. Nurs Health Sci. (2020) 22(2):184–96. 10.1111/nhs.12732 32358875

[8] Rhea DJ. Let the kids play: the impact of chaos on academic success. J Kinesiol Wellness. (2021) 10:98–105. 10.56980/jkw.v10i.98

[9] Bento G Dias G. The importance of outdoor play for young children's healthy development. Porto Biomed J. (2017) 2(5):157–60. 10.1016/j.pbj.2017.03.003 32258612 PMC6806863

[10] Benner AD Boyle AE Sadler S. Parental involvement and adolescents’ educational success: the roles of prior achievement and socioeconomic status. J Youth Adolesc. (2016) 45(6):1053–64. 10.1007/s10964-016-0431-4 26847424

[11] Dickey K Castle K Pryor K. Reclaiming play in schools. Child Educ. (2016) 92(2):111–7. 10.1080/00094056.2016.1150742

[12] Heidorn J Heidorn B. Recess reboot: effective planning and implementation strategies for classroom teachers: column editor: anthony Parish. Strategies. (2018) 31(5):48–52. 10.1080/08924562.2018.1491746

[13] Kirby KJ. Comparisons of play, chronic stress levels, and body composition in elementary school children of six public schools. [thesis dissertation]. Texas Christian University (2022).

[14] Farbo DJ Rhea DJ. The effects of a yearlong recess intervention on body fat shifts in elementary-aged children. Int J Child Health Nutr. (2022) 11(2):60–71. 10.6000/1929-4247.2022.11.02.1

[15] Farbo DJ Rhea DJ. A pilot study examining body composition classification differences between body mass index and bioelectrical impedance analysis in children with high levels of physical activity. Front Pediatr. (2021):1304. 10.3389/fped.2021.724053 PMC8634703 34869095

[16] Dankiw KA Tsiros MD Baldock KL Kumar S. The impacts of unstructured nature play on health in early childhood development: a systematic review. PLoS One. (2020) 15(2):e0229006. 10.1371/journal.pone.0229006 32053683 PMC7018039

[17] Ericsson I Karlsson MK. Motor skills and school performance in children with daily physical education in school–a 9-year intervention study. Scand J Med Sci Sports. (2014) 24(2):273–8. 10.1111/j.1600-0838.2012.01458.x 22487170

[18] Hills AP Dengel DR Lubans DR. Supporting public health priorities: recommendations for physical education and physical activity promotion in schools. Prog Cardiovasc Dis. (2015) 57(4):368–74. 10.1016/j.pcad.2014.09.010 25269062

[19] Saunders TJ Gray CE Poitras VJ Chaput JP Janssen I Katzmarzyk PT Combinations of physical activity, sedentary behaviour and sleep: relationships with health indicators in school-aged children and youth. Appl Physiol Nutr Metab. (2016) 41(6):S283–93. 10.1139/apnm-2015-0626 27306434

[20] Freitas DL Lausen B Maia JA Lefevre J Gouveia ÉR Thomis M Skeletal maturation, fundamental motor skills and motor coordination in children 7–10 years. J Sports Sci. (2015) 33(9):924–34. 10.1080/02640414.2014.977935 25649360

[21] Lengkana AS Rahman AA Alif MN Mulya G Priana A Hermawan DB. Static and dynamic balance learning in primary school students. Int J Hum Move Sports Sci. (2020) 8(6):469–76. 10.13189/saj.2020.080620

[22] Yu W Cha S Seo S. The effect of ball exercise on the balance ability of young adults. J Phys Ther Sci. (2017) 29(12):2087–9. 10.1589/jpts.29.2087 29643579 PMC5890205

[23] Steinberg N Nemet D Pantanowitz M Eliakim A. Gait pattern, impact to the skeleton and postural balance in overweight and obese children: a review. Sports. (2018) 6(3):75. 10.3390/sports6030075 30065150 PMC6162717

[24] Pangrazi RP Beighle A. Dynamic physical education for elementary school children. Human Kinetics Publishers (2019).

[25] Vandorpe B Vandendriessche J Lefèvre J Pion J Vaeyens R Matthys S The *Körperkoordinationstest für Kinder*: reference values and suitability for 6–12-year-old children in flanders. Scand J Med Sci Sports. (2011) 21(3):378–88. 10.1111/j.1600-0838.2009.01067.x 20136753

[26] Dourou E Komessariou A Riga V Lavidas K. Assessment of gross and fine motor skills in preschool children using the peabody developmental motor scales instrument. Eur Psychomotricity J. (2017) 9(1):89–113.

[27] Bruininks RH Bruininks BD. Bruininks-Oseretsky test of motor proficiency (1978).

[28] Iivonen S Sääkslahti A Laukkanen A. A review of studies using the *Körperkoordinationstest für Kinder* (KTK). Eur J Adapt Phys Act. (2016) 8.

[29] Asunta P Viholainen H Ahonen T Rintala P. Psychometric properties of observational tools for identifying motor difficulties–a systematic review. BMC Pediatr. (2019) 19(1):1–13. 10.1186/s12887-019-1657-6 31493795 PMC6731620

[30] Fransen J D’Hondt E Bourgois J Vaeyens R Philippaerts RM Lenoir M. Motor competence assessment in children: convergent and discriminant validity between the BOT-2 short form and KTK testing batteries. Res Dev Disabil. (2014) 35(6):1375–83. 10.1016/j.ridd.2014.03.011 24713517

[31] Nascimento WMD Henrique NR Marques MDS. KTK motor test: review of the main influencing variables. Rev Paul Pediatr. (2019) 37:372–81. 10.1590/1984-0462/;2019;37;3;00013 31241688 PMC6868552

[32] Kiphard EJ Schilling F. Körperkoordinationstest für Kinder. Weinham: Belz test (1974). 5511840

[33] Kiphard EJ Schilling F. Körperkoordinationstest für Kinder 2, überarbeitete und ergänzte Aufgabe. Weinham: Beltz test (2007).

